# Mitochondrial Translocation of High Mobility Group Box 1 Facilitates LIM Kinase 2-Mediated Programmed Necrotic Neuronal Death

**DOI:** 10.3389/fncel.2016.00099

**Published:** 2016-04-13

**Authors:** Hye-Won Hyun, Ah-Reum Ko, Tae-Cheon Kang

**Affiliations:** Department of Anatomy and Neurobiology, Institute of Epilepsy Research, College of Medicine, Hallym UniversityChuncheon, Kangwon-Do, South Korea

**Keywords:** DRP1, epilepsy, leptomycin B, mitochondrial fission, neuronal death, seizure

## Abstract

High mobility group box 1 (HMGB1) acts a signaling molecule regulating a wide range of inflammatory responses in extracellular space. HMGB1 also stabilizes nucleosomal structure and facilitates gene transcription. Under pathophysiological conditions, nuclear HMGB1 is immediately transported to the cytoplasm through chromosome region maintenance 1 (CRM1). Recently, we have reported that up-regulation of LIM kinase 2 (LIMK2) expression induces HMGB1 export from neuronal nuclei during status epilepticus (SE)-induced programmed neuronal necrosis in the rat hippocampus. Thus, we investigated whether HMGB1 involves LIMK2-mediated programmed neuronal necrosis, but such role is not reported. In the present study, SE was induced by pilocarpine in rats that were intracerebroventricularly infused with saline, control siRNA, LIMK2 siRNA or leptomycin B (LMB, a CRM1 inhibitor) prior to SE induction. Thereafter, we performed Fluoro-Jade B staining, western blots and immunohistochemical studies. LIMK2 knockdown effectively attenuated SE-induced neuronal death and HMGB1 import into mitochondria accompanied by inhibiting nuclear HMGB1 release and abnormal mitochondrial elongation. LMB alleviated SE-induced neuronal death and nuclear HMGB1 release. However, LMB did not prevent mitochondrial elongation induced by SE, but inhibited the HMGB1 import into mitochondria. The efficacy of LMB was less effective to attenuate SE-induced neuronal death than that of LIMK2 siRNA. These findings indicate that nuclear HMGB1 release and the subsequent mitochondrial import may facilitate and deteriorate programmed necrotic neuronal deaths. The present data suggest that the nuclear HMGB1 release via CRM1 may be a potential therapeutic target for the programmed necrotic neuronal death induced by SE.

## Introduction

In various neurological diseases, neuronal death simultaneously exhibits the combined features of apoptosis and necrosis (van Lookeren Campagne and Gill, 1996; Unal-Cevik et al., 2004). Therefore, anti-apoptotic or anti-necrotic agents are not sufficient to prevent neuronal death following various insults. This is because multiple cellular events, including excitotoxicity, mitochondrial dysfunction and oxidative stress are complicated in the process of neuronal death (McNaught and Olanow, 2006; Martinez-Vicente et al., 2008; Zhou et al., 2008).

High mobility group box 1 (HMGB1), a non-histone DNA-binding protein, stabilizes nucleosomal structure and facilitates gene transcription (Bustin, 1999; Ellwood et al., 2000; Verrijdt et al., 2002). Under pathophysiological conditions, nuclear HMGB1 is immediately transported to the cytoplasm undergoing necrosis through a nuclear protein exporter, chromosome region maintenance 1 (CRM1; Scaffidi et al., 2002; Faraco et al., 2007). In extracellular release, HMGB1 acts as a signaling molecule regulating a wide range of inflammatory responses by binding to toll-like receptor 4 (TLR4) and/or receptor for advanced glycan endprotducts (RAGE; Wang et al., 1999; Abraham et al., 2000; Scaffidi et al., 2002; Bonaldi et al., 2003; Ditsworth et al., 2007; Maroso et al., 2010). Interestingly, HMGB1 induces a cell death, which is distinct from apoptosis and autophagy, possibly via a specialized necrotic mechanism accompanied by giant mitochondrial formation (Gdynia et al., 2010). Although nuclear HMGB1 release is an indicative for necrotic process and induces inflammatory responses via extracellular events, the role of its release in neuronal death is still unknown.

Recently, we have reported that LIM Kinase 2 (LIMK2, one of F-actin regulators) is involved in programmed necrotic neuronal death induced by status epilepticus (SE, prolonged seizure activity; Kim et al., 2014; Ko et al., 2015). Briefly, SE-induced endothelin-1 expression/release elevates LIMK2 expression independent of caspases and receptor interaction protein 1 (RIP1), which subsequently impairs dynamin-related protein-1 (DRP1)-mediated mitochondrial dynamics, and finally leads to programmed necrosis. Indeed, LIMK2 knockdown and rescue of DRP1 function protected neurons from SE insults. During this process, HMGB1 is released from nuclei undergoing LIMK2-mediated programmed neuronal necrosis, which is inhibited by LIMK2 knockdown (Kim et al., 2014). These findings indicate that HMGB1 may involve programmed neuronal necrosis induced by SE, but such role is not reported. Therefore, we investigated whether HMGB1 involves LIMK2-mediated programmed neuronal necrosis. In the present study, SE resulted in the nuclear HMGB1 release in CA1 neurons. LIMK2 knockdown effectively attenuated SE-induced neuronal death accompanied by inhibiting nuclear HMGB1 release and abnormal mitochondrial elongation. Furthermore, LIMK2 knockdown ameliorated HMGB1 import into mitochondria following SE. In contrast to LIMK2 knockdown, leptomycin B (LMB), a CRM1 inhibitor, did not prevent SE-induced mitochondrial elongation, but alleviated neuronal death, nuclear HMGB1 release and transport of HMGB1 into mitochondria. These findings indicate that nuclear HMGB1 release and the subsequent mitochondrial transport may facilitate or deteriorate programmed necrotic neuronal deaths. Therefore, we suggest that nuclear HMGB1 export may be one of potential therapeutic strategies for LIMK2-mediated programmed necrotic neuronal death.

## Materials and Methods

### Experimental Animals and Chemicals

We used male Sprague-Dawley (SD) rats (7 week old) obtained from Daehan biolink (South Korea). Animals were housed four per cage in a room maintained under 22 ± 2°C, 55 ± 5% and a 12:12 light/dark cycle conditions, and were allowed free access to food and water. All experiments were approved by the Institutional Animal Care and Use Committee of the Hallym University (Chunchon, Republic of Korea). All reagents were obtained from Sigma-Aldrich (St. Louis, MO, USA), unless otherwise noted.

### Surgery, Drug Infusion and LIMK2 Knockdown

Rats were anesthetized with 1–2% Isoflurane in O_2_ and placed in a stereotaxic frame. A brain infusion kit 1 (Alzet, CA, USA) was implanted into the right lateral ventricle (1 mm posterior; 1.5 mm lateral; 3.5 mm depth) and connected to an osmotic pump (1007D, Alzet, CA, USA) containing: (1) control siRNA; (2) LIMK2 siRNA; (3) vehicle; and (4) LMB (30 ng/μl; Kim et al., 2014; Ko et al., 2015). The LIMK2 siRNA sequence (5′→3′) was as followed: sense, GCACCUUACGCAAGAGUGAUU; antisense, UCACUCUUGCGUAAGGUGCUU. A non silencing RNA was used as the control siRNA. In pilot study and our previous studies (Kim et al., 2014; Ko et al., 2015), siRNA or LMB infusion did not affect seizure threshold, seizure activity and BBB integrity. The pump was subcutaneously placed subcutaneously in the interscapular region. Three days after surgery, animals were used for SE induction.

### Seizure Induction

Twenty min before SE induction, animals were injected with methylscopolamine (5 mg/kg, i.p.) followed by pilocarpine (380 mg/kg, i.p.). Two hours after onset of SE, seizure activity was controlled by diazepam (10 mg/kg, i.p.). At designated time courses, animals were used for Immunohistochemistry and western blot. As controls, age-matched normal rats were treated with saline instead of pilocarpine.

### Tissue Processing

Tissue process was performed as described in our previous studies (Kim et al., 2014; Ko et al., 2015). Briefly, animals were perfused transcardially with 4% paraformaldehyde in 0.1 M phosphate buffer (PB, pH 7.4) under urethane anesthesia (1.5 g/kg, I.P.). Brains were removed, postfixed, cryoprotected and sectioned at 30 μm with a cryostat at 30 μm. For western blot, tissues were homogenized and the protein concentration in the supernatant was determined using a Micro BCA Protein Assay Kit (Pierce Chemical, Rockford, IL, USA).

### Immunohistochemistry

Free-floating coronal sections were incubated in a mixture of primary antisera (Table 1) in phosphate-buffered saline (PBS) containing 0.3% Triton X-100 overnight at room temperature and subsequently reacted with a mixture of FITC- and Cy3-conjugated IgG (or streptavidin, Jackson Immunoresearch Laboratories Inc., West Grove, PA, USA). Negative controls were obtained by omitting primary antibody to verify the specificity of the antibodies. Images were captured using an Axiocam HRc camera and AxioVision Rel. 4.8 Software or a confocal laser scanning microscope (LSM 510 META, Carl Zeiss Inc, Oberkocken, Germany). Fluorescent intensity was measured, and standardized by setting the threshold level (mean background intensity obtained from five image input). Manipulation of the images was restricted to threshold and brightness adjustments to the whole image. Individual mitochondrion length in CA1 pyramidal cells (*n* = 20/section) was also measured with a 100× objective lens by AxioVision Rel. 4.8 Software.

**Table 1:** **Primary antibodies used in the present study**.

Antibody	Host	Manufacturer (catalog number)	Dilution used
DRP1	Rabbit	Thermo (PA1–16987)	1:1000 (WB)
DRP1 S616	Rabbit	Cell signaling (#4867)	1:1000 (WB)
DRP1 S637	Rabbit	Cell signaling (#4494)	1:1000 (WB)
HMGB1	Rabbit	Abcam (ab18256)	1:100 (IF)
LIMK2	Rabbit	Abcam (ab45165)	1:100 (IF)
			1:2000 (WB)
Mitochondrial marker	Mouse	Millipore (MAB3494)	1:50 (IF)
NeuN	Mouse	Millipore (MAB377)	1:500(IF)

*IF, Immunofluorescence; WB, Western blot*.

### Fluoro-Jade B Staining

The sections mounted on gelatin-coated slides were immersed in a solution containing 1% sodium hydroxide in 80% ethanol for 5 min and then, 70% ethanol for 2 min and distilled water for 2 min. The slides were then incubated in a solution of potassium permanganate for 15 min and subsequently in 0.001% FJB (Histo-Chem Inc. Jefferson, AR, USA). After staining, the slides were rinsed, dehydrated and mounted DPX. Two different investigators performed cell counts with optical dissector methods (Kim et al., 2014; Ko et al., 2015).

### TUNEL Staining

TUNEL staining was performed with the TUNEL apoptosis detection kit (Merck Millipore, Bedford, MA, USA) according to the manufacturer’s instructions. Following TUNEL reaction, immunofluorescence staining for mitochondrial marker was performed. For nuclei counterstaining, we used Vectashield mounting medium with DAPI (Vector).

### Western Blot

Aliquots were loaded into a polyacrylamide gel. After electrophoresis, gels were transferred to nitrocellulose transfer membranes. Membranes were incubated with primary antibody (Table 1), and visualized by an ECL Kit (Amersham). Intensity measurements were represented as the mean gray-scale value on a 256 gray-level scale. All results were normalized against β-Actin (1:2000; Sigma).

### Data Analysis

One-way ANOVA was applied to determine statistical significance. Bonferroni’s test was used for *post hoc* comparisons. A *p*-value below 0.05 was considered statistically significant.

## Results

### SE Rapidly Increases LIMK2 Expression in CA1 Neurons Vulnerable to SE

**Figure 1:**
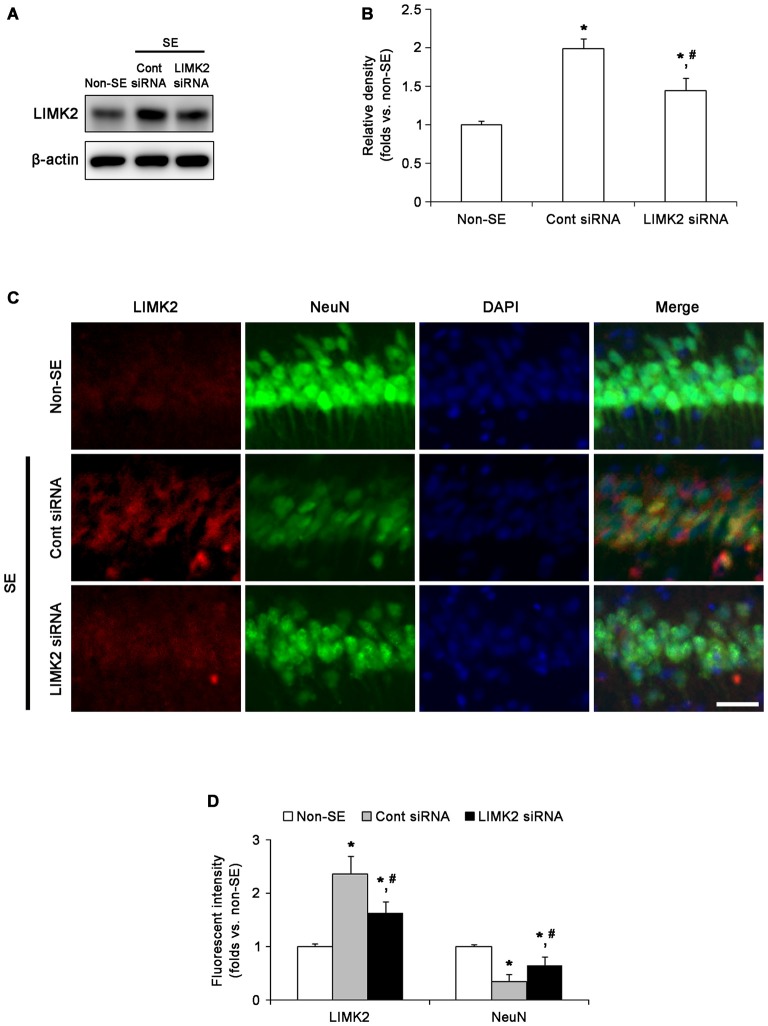
**Effect of LIM Kinase 2 (LIMK2) knockdown on status epilepticus (SE)-induced alterations in LIMK2 protein expression at 3 days after SE. (A)** Western blot image of LIMK2 protein expression in the hippocampus. As compared to control siRNA, LIMK2 siRNA infusion effectively inhibits up-regulation of LIMK2 expression following SE. **(B)** Quantitative values (mean ± SEM) of LIMK2 expression level in the hippocampus, based on western blot (*n* = 7, respectively). *p < 0.05 vs. non-SE; ^#^*p* < 0.05 vs. control siRNA. **(C)** Representative photographs of LIMK2 and NeuN in CA1 neurons. Following SE, LIMK2 expression is elevated in CA1 neurons. LIMK2 siRNA attenuates SE-induced LIMK2 induction in CA1 neurons. Scale bar = 25 μm. **(D)** Quantitative values (mean ± SEM) of LIMK2 and NeuN expression intensity in CA1 neurons, based on immunofluorescent images (*n* = 7, respectively). *p < 0.05 vs. non-SE; ^#^*p* < 0.05 vs. control siRNA.

Consistent with our previous report (Kim et al., 2014), LIMK2 expression in the hippocampus was increased to 1.99-fold of non-SE level 3 days after SE (*p* < 0.05 vs. non-SE animals, Figures 1A,B). LIMK2 siRNA effectively inhibited SE-mediated LIMK2 induction in the hippocampus (*p* < 0.05 vs. control siRNA, Figures 1A,B). Immunohistochemical study revealed that LIMK2 expression was increased to 2.37-fold of non-SE level in the CA1 pyramidal cells 3 days after SE (*p* < 0.05 vs. non-SE animals, Figures 1C,D), while intensity of NeuN expression was reduced 0.35-fold of non-SE level in this region (*p* < 0.05 vs. non-SE animals, Figures 1C,D). LIMK2 siRNA infusion attenuated up-regulation of LIMK2 induction and decrease in NeuN expression induced by SE (*p* < 0.05 vs. control siRNA, Figures 1C,D). LMB infusion did not affect LIMK2 expression in CA1 pyramidal cells 3 days after SE (data not shown). These findings indicate that up-regulation of LIMK2 expression may play an important role in the SE-induced CA1 neuronal death, and nuclear HMGB1 release may not be involved in up-regulation of LIMK2 expression induced by SE.

### LIMK2 siRNA and LMB Inhibit Nuclear HMGB1 Release Induced by SE

**Figure 2:**
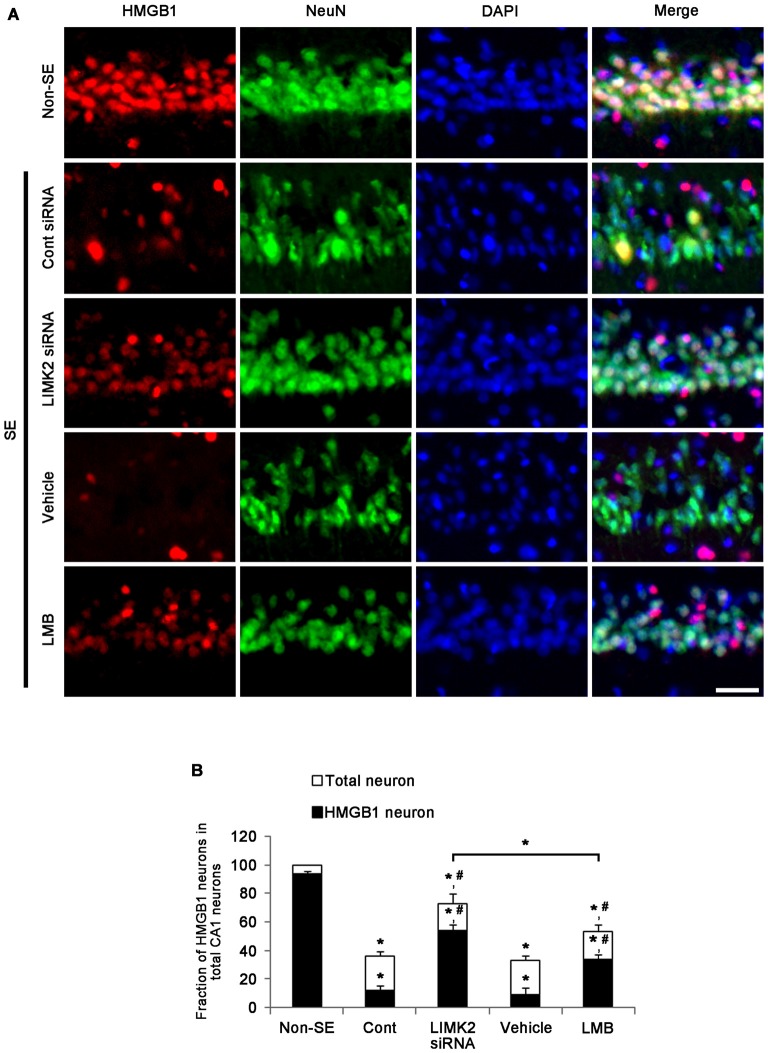
**Effect of LIMK2 knockdown and LMB on SE-induced nuclear high mobility group box 1 (HMGB1) release at 3 days after SE. (A)** Representative photographs of HMGB1 and NeuN in CA1 neurons. Following SE, nuclear HMGB1 expression is reduced in CA1 neurons. Both LIMK2 siRNA and LMB attenuate SE-induced nuclear HMGB1 release and loss of NeuN expression in CA1 neurons, as compared to control siRNA and vehicle, respectively. The effect of LINK2 siRNA is higher than that of LMB. Scale bar = 25 μm. **(B)** Quantitative values (mean ± SEM) of the fraction of HMGB1 neurons in total CA1 neurons (*n* = 7, respectively). *p < 0.05 vs. non-SE; ^#^*p* < 0.05 vs. control siRNA or vehicle, respectively.

Since translocation of HMGB1 from the nucleus to the cytoplasm indicates the necrotic degeneration of various cells (Scaffidi et al., 2002; Faraco et al., 2007; Kim et al., 2014), we investigated whether LIMK2 knockdown or LMB affects SE-induced nuclear HMGB1 export induced by SE. In non-SE animals, HMGB1 expression was restricted to nuclei in CA1 neurons, and the fraction of HMGB1 positive neurons in total CA1 neurons was 94% (Figures 2A,B). Three days after SE, the number of total CA1 neurons was decreased to 36% of non-SE animals, and the fraction of HMGB1 positive neurons in total CA1 neurons was also reduced to 12% (*p* < 0.05 vs. non-SE animals, Figures 2A,B). Both LIMK2 siRNA and LMB effectively attenuated nuclear HMGB1 release and neuronal death induced by SE (*p* < 0.05 vs. control siRNA and vehicle, respectively, Figures 2A,B), but LIMK2 siRNA was more effective than LMB (*p* < 0.05, Figures 2A,B). These findings indicate that nuclear HMGB1 export may be involved in SE-induced neuronal death.

### Inhibition of Nuclear HMGB1 Release Attenuates SE-Induced Programmed Neuronal Necrosis

**Figure 3:**
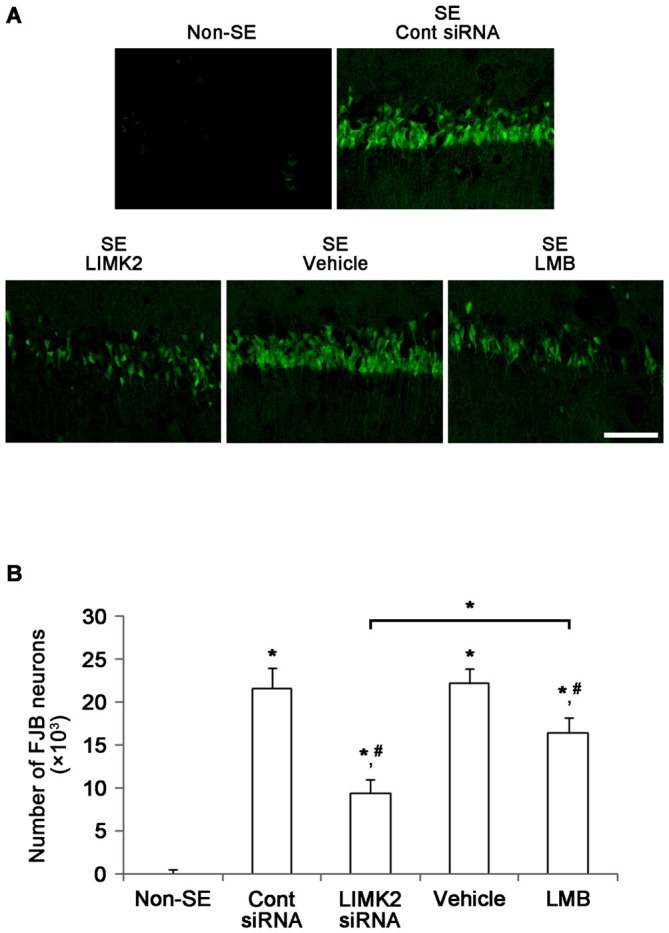
**Effect of LIMK2 knockdown and LMB on SE-induced neuronal damage in the CA1 at 3 days after SE. (A)** FJB-positive neuronal damage in the CA1 at 3 days after SE. Both LIMK2 siRNA and LMB attenuate attenuates SE-induced neuronal damage. However, LIMK2 is more effective than LMB. Scale bar = 50 μm. **(B)** Quantitative values (mean ± SEM) of the number of FJB-positive degenerating neurons (*n* = 7, respectively). *p < 0.05 vs. non-SE; ^#^*p* < 0.05 vs. control siRNA or vehicle, respectively.

Next, we directly confirmed the effects of LIMK2 knockdown and LMB on SE-induced neuronal death. Fluoro-Jade B (FJB) staining showed a prominent loss of neurons in the CA1 region of the hippocampus 3 days after SE (*p* < 0.05 vs. non-SE animals, Figures 3A,B). Consistent with reduction in NeuN expression, LIMK2 siRNA effectively reduced the number of FJB positive neurons induced by SE (*p* < 0.05 vs. control siRNA, Figures 3A,B). LMB also attenuated SE-induced neuronal death, but its efficacy was lower than that of LIMK2 siRNA (*p* < 0.05 vs. vehicle and LIMK2 siRNA, respectively, Figures 3A,B). The present data suggest that nuclear HMGB1 release may exacerbate neuronal death induced by SE.

### Nuclear HMGB1 Release Does not Affect Mitochondrial Elongation Induced by SE

**Figure 4:**
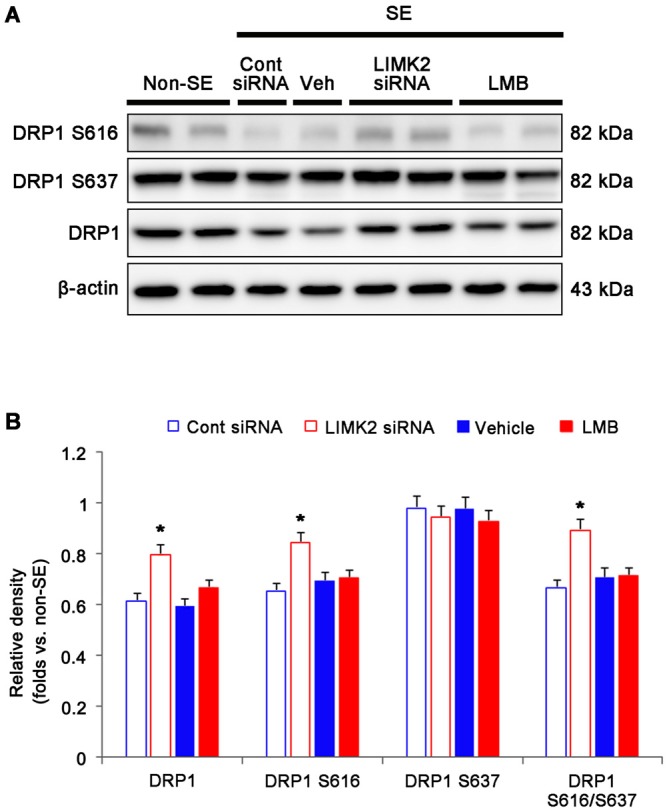
**Effect of LMIK2 siRNA and LMB on HMGB1 expression, DRP1 expression and DRP phosphorylation level at 3 days after SE. (A)** Western blot images of HMGB1, DRP1, DRP1 S616 and DRP1 S637 in the hippocampus. Both LIMK2 siRNA and LMB abolish the reduction of HMGB1 release induced by SE. Only LIMK2 siRNA prevents reductions in DRP1 expression, DRP1 S616 level and DRP1 S616/S637 level induced by SE. **(B)** Quantitative values (mean ± SEM) of HMGB1, DRP1, DRP1 S616, DRP1 S637 level, based on western blot (*n* = 7, respectively). *p < 0.05 vs. control siRNA and vehicle, respectively.

DRP1 is a soluble cytosolic protein that regulates mitochondrial fission. Interestingly, DRP1-S616 phosphorylation accelerates mitochondrial fission, while DRP1-S637 inhibits it. In addition, the dysfunction of mitochondrial dynamics induces necrosis (Kashatus et al., 2011; DuBoff et al., 2012; Wang et al., 2012). Since LIMK2 inhibits mitochondrial fission during SE-induced programmed necrotic cell death (Kim et al., 2014; Ko et al., 2015), we investigated whether HMGB1 translocation is relevant to dysfunction of mitochondrial dynamics. SE decreased DRP1 expression, DRP1 S616 phosphorylation and DRP1 S616/S637 phosphorylation ratio (*p* < 0.05 vs. non-SE animals, Figure 4). LIMK2 siRNA effectively attenuated the reductions in DRP1 expression, DRP1 S616 phosphorylation and DRP1 S616/S637 phosphorylation ratio (*p* < 0.05 vs. control siRNA, Figure 4). However, LMB did not affect DRP1 expression, DRP1 S616 phosphorylation and DRP1 S616/S637 phosphorylation ratio induced by SE (*p* < 0.05 vs. vehicle, Figure 4). These findings indicate that nuclear HMGB1 export may not result in dysfunction of mitochondrial fission induced by SE.

### Mitochondrial HMGB1 Translocation Aggravates SE-Induced Neuronal Death

**Figure 5:**
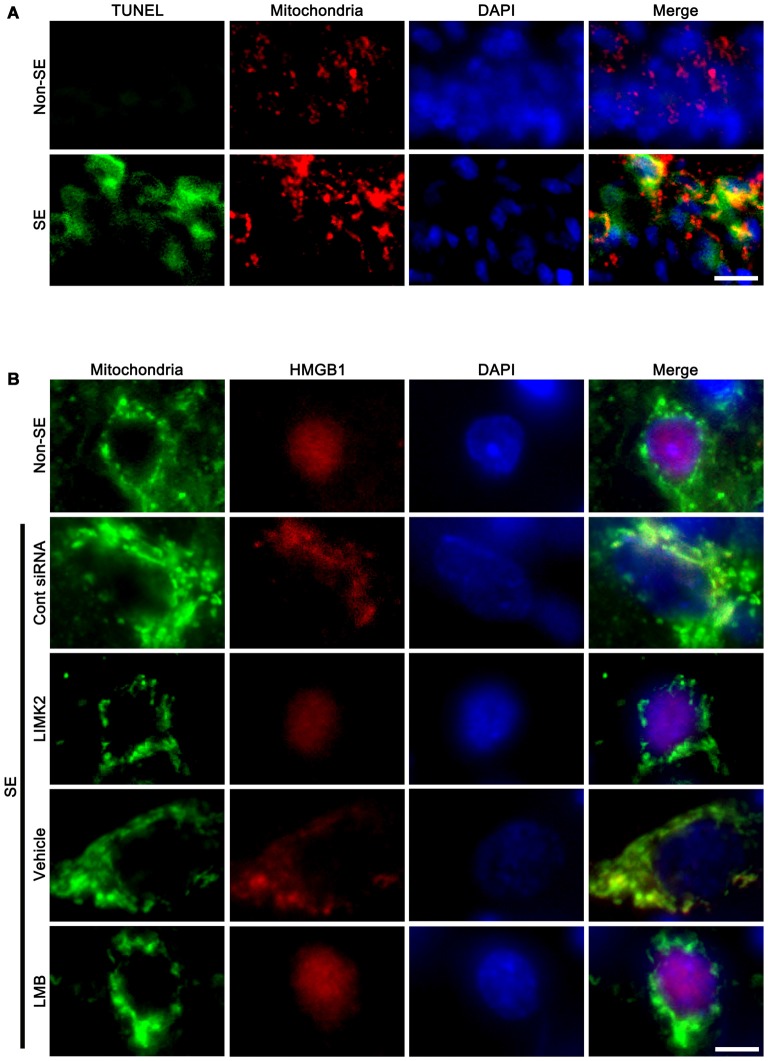
**Mitochondrial elongation in degenerating neurons and the effect of LMIK2 siRNA and LMB on nuclear HMGB1 export at 3 days after SE. (A)** Representative photographs of mitochondrial elongation in TUNEL positive CA1 neurons. Scale bar = 25 μm. **(B)** Representative photographs of HMGB1 and mitochondria in CA1 neurons. SE increases nuclear HMGB1 export and mitochondrial length. LIMK2 siRNA alleviates mitochondrial elongation and nuclear HMGB1 export following SE. LMB attenuates nuclear HMGB1 export, but not mitochondrial elongation induced by SE. Scale bar = 6.25 μm.

To confirm the role of mitochondrial elongation in SE-induced neuronal death, we performed double immunofluorescent study for mitochondrial marker and TUNEL. Following SE, mitochondrial elongation was predominantly observed in TUNEL-positive CA1 neurons (Figure 5A). These findings indicate that mitochondrial elongation may play an important role in programmed neuronal necrosis induced by SE.

**Figure 6:**
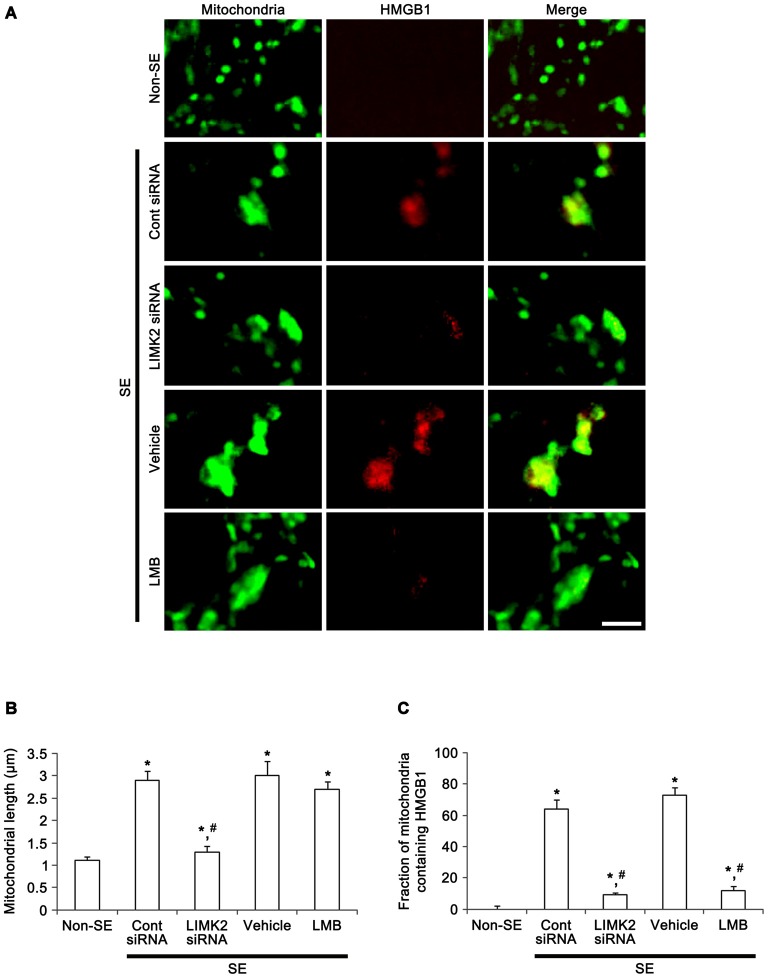
**Effect of LMIK2 siRNA and LMB on mitochondrial HMGB1 import and mitochondrial elongation at 3 days after SE. (A)** Representative photographs of HMGB1 and mitochondria in CA1 neurons. SE increases mitochondrial HMGB1 import as well as mitochondrial elongation. LIMK2 siRNA alleviates mitochondrial elongation and mitochondrial HMGB1 import. LMB attenuates mitochondrial HMGB1 import, but not mitochondrial elongation induced by SE. Scale bar = 3 μm. **(B)** Quantitative values (mean ± SEM) of mitochondrial length in CA1 neurons (*n* = 7, respectively). *p < 0.05 vs. non-SE; ^#^*p* < 0.05 vs. control siRNA or vehicle, respectively. **(C)** Quantitative values (mean ± SEM) of the fraction of mitochondria containing HMGB1 in total mitochondria (*n* = 7, respectively). *p < 0.05 vs. non-SE; ^#^*p* < 0.05 vs. control siRNA or vehicle, respectively.

In addition, SE increased mitochondrial length and sphere formation in CA1 neurons accompanied by nuclear HMGB1 export. LIMK2 siRNA effectively abolished mitochondrial elongation and HMGB1 translocation into cytoplasm induced by SE. LMB also significantly inhibited HMGB1 export, but did not prevent mitochondrial elongation (Figure 5B). Confocal microscopic analysis also revealed the SE induced mitochondrial elongation and HMGB1 imports into mitochondria (*p* < 0.05 vs. non-SE animals, Figures 6A–C). LIMK2 siRNA effectively attenuated mitochondrial elongation and HMGB1 translocation into mitochondria induced by SE (*p* < 0.05 vs. control siRNA, Figures 6A–C). LMB did not prevent mitochondrial elongation (Figures 6A–C), but significantly inhibited mitochondrial HMGB1 translocation (*p* < 0.05 vs. control siRNA, Figures 6A–C). Taken together, our findings indicate that nuclear HMGB1 export and the subsequent mitochondrial translocation may facilitate and exacerbate programmed neuronal necrosis induced by SE.

## Discussion

Recently, we have reported that LIMK2 induces a novel form of programmed neuronal necrosis that is characterized by the dysfunction of mitochondrial fission and nuclear HMGB1 release (Kim et al., 2014). This SE-induced programmed necrosis is independent of RIP1, since necrostatin-1 (a RIP1 inhibitor) cannot attenuate LIMK2-mediated neuronal death. Furthermore, LIMK2 knockdown alleviates SE-induced neuronal death accompanied by inhibition of nuclear HMGB1 release (Kim et al., 2014). Consistent with our previous report, the present data demonstrate that SE increased mitochondrial length and sphere formation in CA1 neurons accompanied by decrease in DRP1 S616/S637 phosphorylation ratio. LIMK2 siRNA also effectively abolished SE-induced mitochondrial elongation and sphere formation and attenuated the reduction in DRP1 S616/S637 phosphorylation ratio. Some previous studies reported that this aberrant mitochondrial fission plays an important roles in neuronal degeneration, since elongated mitochondria disrupt their translocations to dendrites or axons, and in turn suppress ATP supply in peripheral sites (Li et al., 2004; Verstreken et al., 2005). Elongated mitochondria also show the impaired respiratory function that leads to excessive reactive oxygen species production and a decrease in ATP synthesis (Parone et al., 2008; Kashatus et al., 2011; Kageyama et al., 2012). Indeed, the present study demonstrates that mitochondrial elongation was predominantly detected in TUNEL positive neurons. Furthermore, DRP1 over-expression increases neuronal viability by activation of mitochondrial fission following DNA damage (Wang et al., 2013), and the rescue of dysfunction of mitochondrial fission effectively prevents nuclear HMGB1 release induced by SE (Kim et al., 2014). Therefore, it is likely that impaired mitochondrial fission induced by LIMK2 may provoke neuronal necrosis, which in turn can induce nuclear HMGB1 release following SE.

Traditionally, HMGB1 is released from damaged or dying cells during necrosis into the extracellular space (Qiu et al., 2008), subsequently it evokes potentially inflammatory responses via RAGE or TLR4 (Abraham et al., 2000; Andersson et al., 2000; Scaffidi et al., 2002; Park et al., 2004; Kim et al., 2006; Maroso et al., 2010). Consistent with our recent study (Kim et al., 2014), the present data show that SE increased LIMK2 expression in CA1 neurons accompanied by nuclear HMGB1 release. Furthermore, LMB effectively attenuated nuclear HMGB1 release and neuronal death induced by SE, although it could not prevent SE-induced LIMK2 induction. These findings indicate that nuclear HMGB1 release may not be only an indicative for neuronal necrosis representing nonspecific leakage from damaged nucleus or neuroinflammation, but also may participate in SE-induced programmed necrotic neuronal death.

What is the role of nuclear HMGB1 export in SE-induced programmed necrotic neuronal death? Ito et al. (2014) reported that HMGB1 rescues the impairment of mitochondrial function. In endothelial cells, the translocation of endogenous HMGB1 from the nucleus to the mitochondria involves mitochondrial reorganization (Stumbo et al., 2008). In cancer cells, exogenous HMGB1 enters the mitochondria, which is followed by the formation of giant mitochondria independently of HMGB1 receptors such as TLR4 or RAGE (Gdynia et al., 2010). Therefore, it is likely that the nuclear HMGB1 export would be involved in aberrant mitochondrial fission or the compensatory responses for the maintenance of mitochondrial functions. However, the present study demonstrates that LMB-mediated inhibition of nuclear HMGB1 export could not affect mitochondrial elongation, sphere formation and the reduction in DRP1 S616/S637 phosphorylation ratio. Hence, these findings suggest that at least in neurons nuclear HMGB1 export may not be involved in mitochondrial dynamics. Conversely, the present study shows that LMB attenuated SE-induced neuronal death accompanied by abolishing the mitochondrial HMGB1 import, although it could not inhibit mitochondrial elongation following SE. These findings suggest that the translocation of HMGB1 into elongated mitochondria may aggravate or facilitate SE-induced neuronal death, although the present data could not provide the exact biological meaning of mitochondrial HMGB1 import for SE-induced neuronal death. To elucidate the role of mitochondrial HMGB1 import in SE-induced neuronal death, further studies are needed.

On the other hand, some investigators reported that Mdivi-1, a selective inhibitor of DRP1, attenuates oxidative stress and reduces neuronal loss after pilocarpine-induced SE (Qiu et al., 2013; Xie et al., 2013). Since any differences in seizure severity or susceptibility will result in the distinct consequences on seizure-induced neuronal death, these discrepancies are resulted from the distinct methodology inducing SE. In the present study, we applied 2 h-lasting SE induction, while other investigators used 1 h-lasting SE method (Qiu et al., 2013; Xie et al., 2013). In our model, indeed, Mdivi-1 (5 and 50 μM) treatment significantly increases SE-induced mitochondrial elongation and the number of dying neurons (Kim et al., 2014). Taken together, our findings indicate that the duration and severity of seizure activity may distinctly influence mitochondrial fragmentation or elongation.

In conclusion, our findings demonstrate that inhibition of nuclear HMGB1 release attenuated SE-induced necrotic neuronal death, although it could not ameliorate disruption of mitochondrial fission. These findings provide a novel role of HMGB1 in LIMK2-mediated programmed neuronal necrosis. Therefore, the present data suggest that the nuclear HMGB1 release via CRM1 may be a potential therapeutic target for the programmed necrotic neuronal death induced by SE.

## Author Contributions

T-CK designed the study; H-WH, A-RK performed the experiments. H-WH, A-RK and T-CK analyzed the data and wrote the article. H-WH, A-RK and T-CK read and approved the final manuscript.

## Conflict of Interest Statement

The authors declare that the research was conducted in the absence of any commercial or financial relationships that could be construed as a potential conflict of interest.

## Abbreviations

CRM1: chromosome region maintenance 1
DRP1: dynamic related protein-1
FJB: fluoro-Jade B
HMGB1: high mobility group box 1
IF: Immunofluorescence
LIMK2: LIM kinase 2
LMB: leptomycin B
PB: phosphate buffer
PBS: phosphate-buffered saline
RIP1: receptor interaction protein 1
RAGE: receptor for advanced glycan endprotducts
SD: Sprague-Dawley
SE: status epilepticus
TLR4: toll-like receptor 4
WB: Western blot.

## References

[B1] Abraham E. Arcaroli J. Carmody A. Wang H. Tracey K. J. (2000). HMG-1 as a mediator of acute lung inflammation. J. Immunol. 165, 2950–2954. 10.4049/jimmunol.165.6.2950 10975801

[B2] Andersson U. Wang H. Palmblad K. Aveberger A. C. Bloom O. Erlandsson-Harris H.. (2000). High mobility group 1 protein (HMG-1) stimulates proinflammatory cytokine synthesis in human monocytes. J. Exp. Med. 192, 565–570. 10.1084/jem.192.4.565 10952726 PMC2193240

[B3] Bonaldi T. Talamo F. Scaffidi P. Ferrera D. Porto A. Bachi A.. (2003). Monocytic cells hyperacetylate chromatin protein HMGB1 to redirect it towards secretion. EMBO J. 22, 5551–5560. 10.1093/emboj/cdg516 14532127 PMC213771

[B4] Bustin M. (1999). Regulation of DNA-dependent activities by the functional motifs of the high-mobility-group chromosomal proteins. Mol. Cell. Biol. 19, 5237–5246. 10.1128/mcb.19.8.5237 10409715 PMC84367

[B5] Ditsworth D. Zong W. X. Thompson C. B. (2007). Activation of poly (ADP)-ribose polymerase (PARP-1) induces release of the pro-inflammatory mediator HMGB1 from the nucleus. J. Biol. Chem. 282, 17845–17854. 10.1074/jbc.m701465200 17430886 PMC3140953

[B6] DuBoff B. Götz J. Feany M. B. (2012). Tau promotes neurodegeneration via DRP1 mislocalization *in vivo*. Neuron. 75, 618–632. 10.1016/j.neuron.2012.06.026 22920254 PMC3428596

[B7] Ellwood K. B. Yen Y. M. Johnson R. C. Carey M. (2000). Mechanism for specificity by HMG-1 in enhanceosome assembly. Mol. Cell Biol. 20, 4359–4370. 10.1128/mcb.20.12.4359-4370.2000 10825199 PMC85803

[B8] Faraco G. Fossati S. Bianchi M. E. Patrone M. Pedrazzi M. Sparatore B.. (2007). High mobility group box 1 protein is released by neural cells upon different stresses and worsens ischemic neurodegeneration. J. Neurochem. 103, 590–603. 10.1111/j.1471-4159.2007.04788.x 17666052

[B9] Gdynia G. Keith M. Kopitz J. Bergmann M. Fassl A. Weber A. N.. (2010). Danger signaling protein HMGB1 induces a distinct form of cell death accompanied by formation of giant mitochondria. Cancer Res. 70, 8558–8568. 10.1158/0008-5472.can-10-0204 20959471

[B10] Ito H. Fujita K. Tagawa K. Chen X. Homma H. Sasabe T.. (2014). HMGB1 facilitates repair of mitochondrial DNA damage and extends the lifespan of mutant ataxin-1 knock-in mice. EMBO Mol. Med. 7, 78–101. 10.15252/emmm.201404392 25510912 PMC4309669

[B11] Kageyama Y. Zhang Z. Roda R. Fukaya M. Wakabayashi J. Wakabayashi N.. (2012). Mitochondrial division ensures the survival of postmitotic neurons by suppressing oxidative damage. J. Cell Biol. 197, 535–551. 10.1083/jcb.201110034 22564413 PMC3352955

[B12] Kashatus D. F. Lim K. H. Brady D. C. Pershing N. L. Cox A. D. Counter C. M. (2011). RALA and RALBP1 regulate mitochondrial fission at mitosis. Nat. Cell Biol. 13, 1108–1115. 10.1038/ncb2310 21822277 PMC3167028

[B13] Kim J. B. Sig Choi J. Yu Y. M. Nam K. Piao C. S. Kim S. W.. (2006). HMGB1, a novel cytokine-like mediator linking acute neuronal death and delayed neuroinflammation in the postischemic brain. J. Neurosci. 26, 6413–6421. 10.1523/jneurosci.3815-05.2006 16775128 PMC6674036

[B14] Kim J. E. Ryu H. J. Kim M. J. Kang T. C. (2014). LIM kinase-2 induces programmed necrotic neuronal death via dysfunction of DRP1-mediated mitochondrial fission. Cell Death Differ. 21, 1036–1049. 10.1038/cdd.2014.17 24561342 PMC4207472

[B15] Ko A. R. Hyun H. W. Min S. J. Kim J. E. Kang T. C. (2015). Endothelin-1 induces LIMK2-mediated programmed necrotic neuronal death independent of NOS activity. Mol Brain. 8: 58. 10.1186/s13041-015-0149-3 26438559 PMC4595180

[B16] Li Z. Okamoto K. Hayashi Y. Sheng M. (2004). The importance of dendritic mitochondria in the morphogenesis and plasticity of spines and synapses. Cell 119, 873–887. 10.1016/j.cell.2004.11.003 15607982

[B17] Maroso M. Balosso S. Ravizza T. Liu J. Aronica E. Iyer A. M.. (2010). Toll-like receptor 4 and high-mobility group box-1 are involved in ictogenesis and can be targeted to reduce seizures. Nat. Med. 16, 413–419. 10.1038/nm.2127 20348922

[B18] Martinez-Vicente M. Talloczy Z. Kaushik S. Massey A. C. Mazzulli J. Mosharov EV.. (2008). Dopamine-modified alpha-synuclein blocks chaperone-mediated autophagy. J. Clin. Invest. 118, 777–788. 10.1172/JCI32806 18172548 PMC2157565

[B19] McNaught K. S. Olanow C. W. (2006). Protein aggregation in the pathogenesis of familial and sporadic Parkinson’s disease. Neurobiol. Aging. 27, 530–545. 10.1016/j.neurobiolaging.2005.08.012 16207501

[B20] Park J. S. Svetkauskaite D. He Q. Kim J. Y. Strassheim D. Ishizaka A.. (2004). Involvement of toll-like receptors 2 and 4 in cellular activation by high mobility group box 1 protein. J. Biol. Chem. 279, 7370–7377. 10.1074/jbc.m306793200 14660645

[B21] Parone P. A. Da Cruz S. Tondera D. Mattenberger Y. James D. I. Maechler P.. (2008). Preventing mitochondrial fission impairs mitochondrial function and leads to loss of mitochondrial DNA. PLoS One. 3:e3257. 10.1371/journal.pone.0003257 18806874 PMC2532749

[B22] Qiu X. Cao L. Yang X. Zhao X. Liu X. Han Y. (2013). Role of mitochondrial fission in neuronal injury in pilocarpine-induced epileptic rats. Neuroscience 245, 157–165. 10.1016/j.neuroscience.2013.04.019 23597828

[B23] Qiu J. Nishimura M. Wang Y. Sims J. R. Qiu S. Savitz S. I.. (2008). Early release of HMGB-1 from neurons after the onset of brain ischemia. J. Cereb. Blood Flow Metab. 28, 927–938. 10.1038/sj.jcbfm.9600582 18000511

[B24] Scaffidi P. Misteli T. Bianchi M. E. (2002). Release of chromatin protein HMGB1 by necrotic cells triggers inflammation. Nature 418, 191–195. 10.1038/nature09475 12110890

[B25] Stumbo A. C. Cortez E. Rodrigues C. A. Henriques M. D. Porto L. C. Barbosa H. S.. (2008). Mitochondrial localization of nonhistone protein HMGB1 during human endothelial cell-Toxoplasma gondii infection. Cell Biol. Int. 32, 235–238. 10.1016/j.cellbi.2007.08.031 17936030

[B26] Unal-Cevik I. Kilinç M. Can A. Gürsoy-Ozdemir Y. Dalkara T. (2004). Apoptotic and necrotic death mechanisms are concomitantly activated in the same cell after cerebral ischemia. Stroke 35, 2189–2194. 10.1161/01.str.0000136149.81831.c5 15256676

[B27] van Lookeren Campagne M. Gill R. (1996). Ultrastructural morphological changes are not characteristic of apoptotic cell death following focal cerebral ischaemia in the rat. Neurosci. Lett. 213, 111–114. 10.1016/0304-3940(96)12839-1 8858621

[B28] Verrijdt G. Haelens A. Schoenmakers E. Rombauts W. Claessens F. (2002). Comparative analysis of the influence of the high-mobility group box 1 protein on DNA binding and transcriptional activation by the androgen, glucocorticoid, progesterone and mineralocorticoid receptors. Biochem. J. 361, 97–103. 10.1042/bj3610097 11742533 PMC1222283

[B29] Verstreken P. Ly C. V. Venken K. J. Koh T. W. Zhou Y. Bellen H. J. (2005). Synaptic mitochondria are critical for mobilization of reserve pool vesicles at Drosophila neuromuscular junctions. Neuron 47, 365–378. 10.1016/j.neuron.2005.06.018 16055061

[B30] Wang D. B. Garden G. A. Kinoshita C. Wyles C. Babazadeh N. Sopher B.. (2013). Declines in Drp1 and parkin expression underlie DNA damage-induced changes in mitochondrial length and neuronal death. J. Neurosci. 33, 1357–1365. 10.1523/jneurosci.3365-12.2013 23345212 PMC3711762

[B31] Wang H. Bloom O. Zhang M. Vishnubhakat J. M. Ombrellino M. Che J.. (1999). HMG-1 as a late mediator of endotoxin lethality in mice. Science. 285, 248–251. 10.1126/science.285.5425.248 10398600

[B32] Wang Z. Jiang H. Chen S. Du F. Wang X. (2012). The mitochondrial phosphatase PGAM5 functions at the convergence point of multiple necrotic death pathways. Cell 148, 228–243. 10.1016/j.cell.2011.11.030 22265414

[B33] Xie N. Wang C. Lian Y. Zhang H. Wu C. Zhang Q. (2013). A selective inhibitor of Drp1, mdivi-1, protects against cell death of hippocampal neurons in pilocarpine-induced seizures in rats. Neurosci. Lett. 545, 64–68. 10.1016/j.neulet.2013.04.026 23628672

[B34] Zhou C. Huang Y. Przedborski S. (2008). Oxidative stress in Parkinson’s disease: a mechanism of pathogenic and therapeutic significance. Ann. N. Y. Acad. Sci. 1147, 93–104. 10.1196/annals.1427.023 19076434 PMC2745097

